# Corticospinal excitability after 5-day Dry Immersion in women

**DOI:** 10.3389/fncir.2023.1163346

**Published:** 2023-09-22

**Authors:** Inna Nosikova, Alexandra Riabova, Vladimir Kitov, Elena Tomilovskaya

**Affiliations:** Laboratory of Gravitational Physiology of the Sensorimotor System, Institute of Biomedical Problems of the Russian Academy of Sciences, Moscow, Russia

**Keywords:** dry immersion, microgravity, transcranial magnetic stimulation, trans-spinal magnetic stimulation, support unloading, motor evoked potentials, hypogravitational hyperreflexia

## Abstract

In light of the development of manned astronautics and the increasing participation of women in space flights, the question of female body adaptation to microgravity conditions becomes relevant. Currently, one of the important directions in this issue is to study the effects of support withdrawal as a factor of weightlessness on the human sensorimotor system. Dry Immersion is one of the well-known ground-based models, which adequately reproduces the main physiological effects of space flight. The aim of this study was to evaluate the changes in motor evoked potentials of the lower leg gravity-dependent muscles in women after a 5-day Dry Immersion. We analyzed evoked responses to transcranial and trans-spinal magnetic stimulation. In this method, areas of interest (the motor cortex and lumbosacral thickening of the spinal cord) are stimulated with an electromagnetic stimulus. The experiment was conducted with the participation of 16 healthy female volunteers with a natural menstrual cycle. The thresholds, amplitudes, and latencies of motor potentials evoked by magnetic stimulation were assessed. We showed that 5-day exposure to support withdrawal leads to a decrease in motor-evoked potential thresholds and central motor conduction time, although changes in motor response amplitudes were ambiguous. The data obtained correspond to the results of previous research on Dry Immersion effects on the sensorimotor system in men.

## 1. Introduction

The key problem of long-term space flights (SF) is extreme conditions for the human body. The space crew needs to live and work in this environment for a long time. Additionally, constant dangers and a high probability of unforeseen emergencies may require an instant shift of attention. Therefore, for successful work in these conditions, it is necessary to correctly assess the capabilities of the body as a whole (Grigoriev and Ushakov, 2013). Under the conditions of constant and continuous stressors, a favorable psychological climate on the spacecraft is crucial, and the presence of women will contribute to its formation and maintenance. Even though, at this date, ~600 people have participated in space missions, only 65 of them were women.

In their review article, Mark et al. (2014) noted that the physiological adaptation to weightlessness in women and men is different. For example, the specifics of vascular resistance dynamics, circulating blood volume regulation, and a more pronounced course of space motion sickness are noted in women; however, clinically significant visual and hearing impairments caused by SF are observed only in male astronauts. This study draws attention to the problem that there is a lack of data to narrow down the features of female body adaptation to the conditions of SF (Mark et al., 2014). In another study, the same authors pointed out the sex differences in the musculoskeletal system, which entails the need to consider astronauts' sex when developing countermeasures for long-term and extra-long-term SF (Mark, 2007; Holt et al., 2016).

Previously, for the first time, a comprehensive experiment to study the effects of 3-day support unloading on the sensorimotor system in women of reproductive age was carried out. It showed the possibility and safety of such exposure, as well as the comparability of the results with data obtained in similar experiments with the participation of male volunteers (Amirova et al., 2021; Tomilovskaya et al., 2021; Nosikova et al., 2021a).

The changes in motor function caused by microgravity exposure may affect astronauts' performance, and although there are many studies of the sensorimotor system in SF and model experiments, the mechanisms of the occurring changes are still not fully understood. Thus, research in this field continues to be of high importance. It is known that exposure to real or simulated microgravity leads to the development of hypogravitational motor syndrome (Kozlovskaya et al., 1988), which is defined by both structural and functional alterations in the neuromuscular system. Changes in brain volume, microstructure, and connectivity were observed after SF (Koppelmans et al., 2016; Jillings et al., 2020) and head-down tilt bed rest (Koppelmans et al., 2017; Lee et al., 2021), and the increase in excitability of motor responses was noted during support withdrawal (Zakirova et al., 2015) and parabolic flight (Davey et al., 2004; Badran et al., 2020). At the molecular level, changes in neuromuscular junctions, such as a decline in the postsynaptic excitatory potential amplitudes, a change in the enzyme activity, and a shift in the level of the ion equilibrium potentials, were reported in both animal (Tyapkina et al., 2006; Chibalin et al., 2018; Vilchinskaya et al., 2018) and human studies (Shenkman et al., 2017; Shenkman, 2020).

As it is not possible to use invasive techniques to study the human brain and the spinal cord during SF or under the conditions of support withdrawal, a different method is required. In the past couple of decades, the transcranial magnetic stimulation (TMS) technique has received widespread use in the fields of space medicine and physiology (Davey et al., 2004; Roberts et al., 2010; Badran et al., 2020; Romanella et al., 2020). Besides its contribution to studying cognition, behavior, and neuropathology, TMS of the motor cortex has a well-established role in clinical neurophysiology. In this method, a target area is stimulated with electromagnetic stimuli, which generates suprathreshold current in the brain. Standard TMS parameters that are analyzed in clinical and research studies include motor thresholds, motor evoked potential (MEP) amplitudes, MEP latencies, etc. Each of these variables has physiological correlates; for instance, MEP thresholds reflect the excitability of cortical or spinal neurons, but at the same time, they depend on the individual arousal level and environmental noise. Amplitudes of the evoked responses are often used to study corticospinal excitability, and the difference between MEP latencies to stimulation of the motor cortex and spinal roots, called central motor conduction time (CMCT), is calculated to estimate corticospinal conductibility (Rossini et al., 2015). Moreover, MEPs to TMS depend on physical individual features such as age, body constitution, and height. Regarding sex-specific differences, there is evidence that women show smaller latencies of responses in upper limb muscles to both cortical and spinal stimulation when compared to men (Cantone et al., 2019). Previously, TMS studies in the field of space physiology were conducted in parabolic flight (Davey et al., 2004; Badran et al., 2020) and long-term bedrest (Roberts et al., 2010), but their results are contradictory and the samples are very small. Moreover, there is almost no data on female subjects and how they compare to men.

Given the above, our work was dedicated to the study of changes in the sensorimotor system in women after exposure to simulated microgravity. We chose Dry Immersion (DI) as one of the most widely used ground-based microgravity models, which adequately reproduces the main physiological effects of weightlessness (Tomilovskaya et al., 2019) and conducted a comprehensive experiment to assess the characteristics of motor responses evoked by transcranial and trans-spinal magnetic stimulation in women before and after exposure to 5-day DI.

## 2. Materials and methods

### 2.1. Participants

A total of 16 healthy female volunteers (mean age 28.06 ± 4.85 years) of reproductive age participated in this study. The participants' height did not exceed 180 cm, and their body weight was not more than 75 kg. All subjects had a natural menstrual cycle (MC) and no history of motor impairments or neurological diseases. Each participant signed an informed consent after the experimental procedures, and possible consequential effects and risks were explained to them.

This study was approved by the Bioethical Commission of the Institute of Biomedical Problems of the Russian Academy of Sciences (Protocol No. 615 of 6 June 2022) and fully complied with the principles of the Declaration of Helsinki.

### 2.2. Experimental design

**Figure 1:**
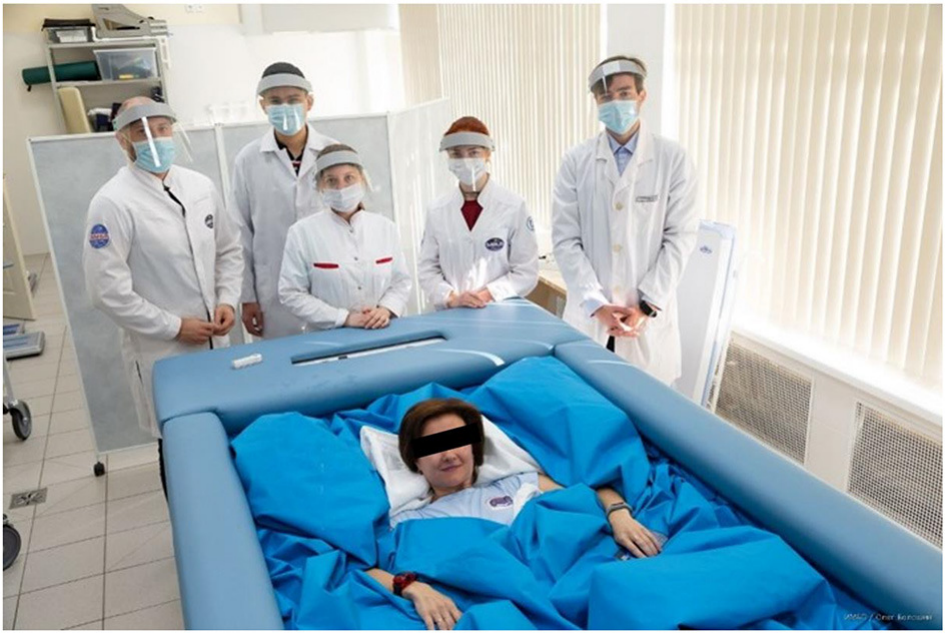
View of a dry immersion bath with the subject and the crew. Image credit: IBMP/Oleg Voloshin.

The study was carried out at the Dry Immersion (DI) facility (Tomilovskaya et al., 2019), which is a part of the unique scientific installation “Medical and technical complex for testing innovative technologies of space biomedicine in the interests of supporting orbital and interplanetary missions and for the development of practical healthcare” of the Institute of Biomedical Problems, Russian Academy of Sciences, and at the unique scientific installation “Transgenbank.” Participants spent 5 days in the immersion bath (Figure 1), with the restriction of motor activity; among other factors, lower limb movements were limited. The water temperature in the bath was kept at the level of 33 ± 1°C. During hygiene procedures scheduled in the evening, the subjects were lifted out of the bath for no longer than 15 min, and most of that time they remained in the supine position. The subjects were also raised from the bath during the day for certain short-term experimental examinations that were also carried out in the supine position. The crew, which included a medical doctor, an assistant, and a technician, provided 24-h monitoring of the participants' health and the condition of the technical equipment. In their free time, subjects were allowed to read, work on a laptop, watch TV, talk on the phone, etc.

Half of the experimental group (nine participants) additionally got the food supplement “Goldoferrin C” starting from the 1st day of DI until the 29th day of the recovery period. “Goldoferrin C” contains human lactoferrin, which is a globular glycoprotein of the transferrin family and is widely present in various secretory liquids such as milk, saliva, tears, and nasal gland secretion. This protein is considered a multifunctional factor in constitutive immunity, tissue differentiation, and cell activation. It also serves as an anabolic factor, promoting bone mass growth. The food supplement “Goldoferrin C” was taken daily: twice a day (in the morning and the evening) during DI and on the day of its completion, then only once a day in the morning. The supplement intake was carried out at least 30 min before the meal. Placebo was used in the control group.

### 2.3. Magnetic stimulation procedure

**Figure 2:**
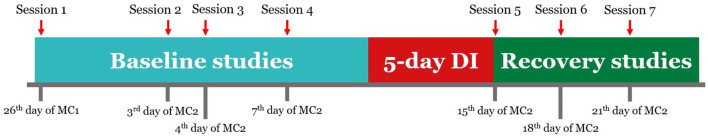
Schedule of the experiment. Sessions 1-4 were conducted 14, 7, 6, and 3 days before the start of DI. Session 5 was conducted on the day of DI completion. Sessions 6 and 7 were conducted 3 and 6 days after DI. MC1, MC2—two consecutive menstrual cycles.

Experimental sessions were conducted according to the schedule (Figure 2). There were seven sessions in total: 4 before DI (14, 7, 6, and 3 days before the start of DI, baseline studies), one on the day of DI completion, referred to as R+0, and 2 during the recovery period (on the 3rd day and the 6th day after DI accomplishment, referred to as R+3 and R+6). Every experimental stage started on a specific day of the MC.

Participants were instructed to abstain from alcoholic and tonic drinks the day before each session. During the procedure, the subjects lay prone, relaxed, and with their eyes open. A special rigid cushion was placed under the ankles for better relaxation. Transcranial and trans-spinal magnetic stimulation (MS) was used to obtain MEPs from the soleus and gastrocnemius (lateral head) muscles of the right leg.

**Figure 3:**
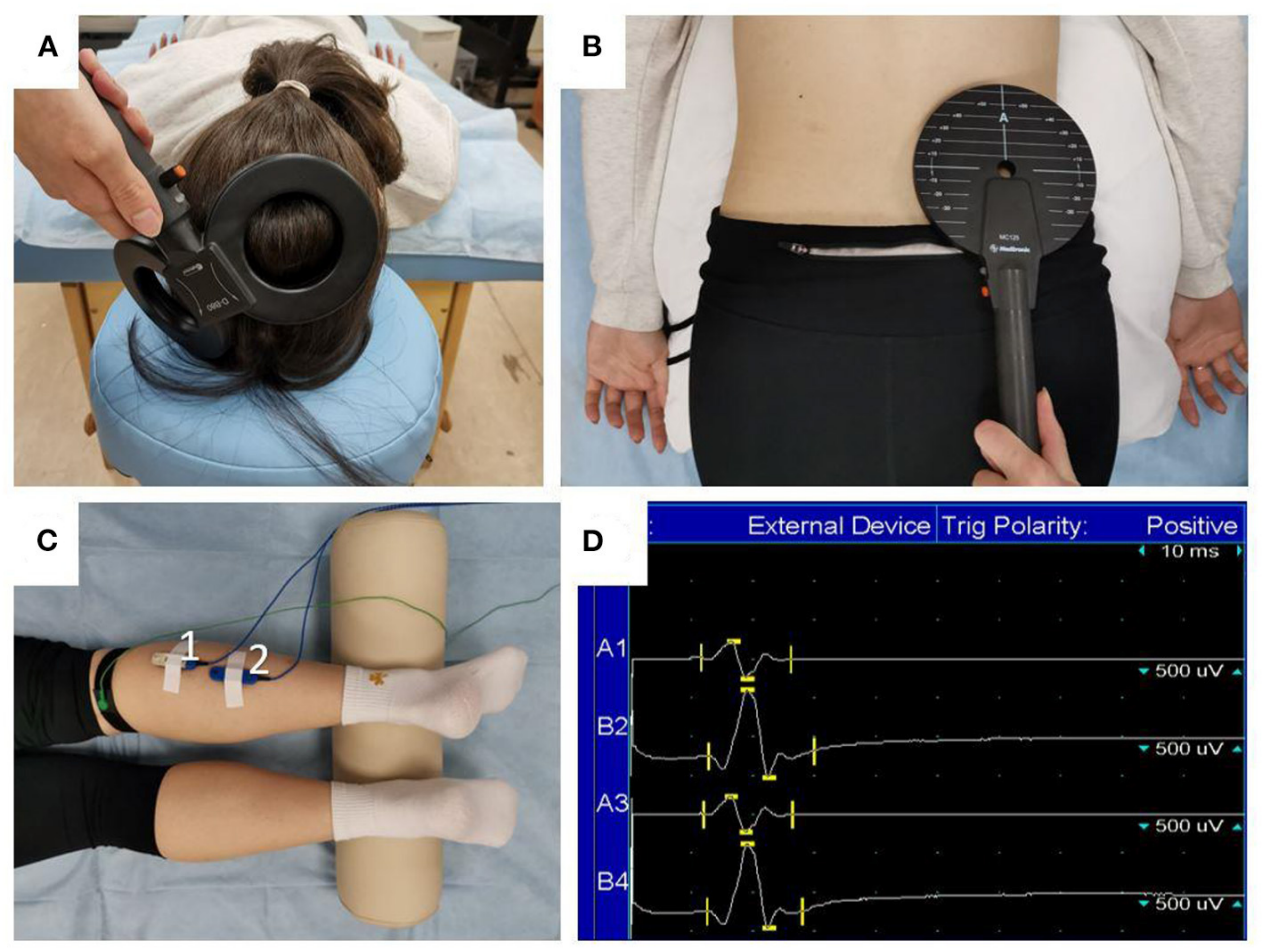
Magnetic stimulation procedure. The position of the stimulator's coil during transcranial **(A)** and trans-spinal **(B)** MS, and the placement of the surface electrodes **(C)** to record MEPs: 1—gastrocnemius muscle (lateral head); 2—soleus muscle. Raw EMG tracings **(D)** are presented for two recordings of MEPs in the gastrocnemius (lines A1 and A3) and soleus (lines B2 and B4) muscles.

Transcranial MS was delivered with the eight-shaped coil (DB-80 Butterfly) of the MagPro X100 magnetic stimulator (Medtronic, Denmark). Stimuli were directed to the area of cortical motor projections of the right lower leg muscles (Figure 3A). First, the intersection point of the vertex and the line connecting the pre-auricular points was determined, and then the coil was placed 1–2 cm to the left from that point and was gradually moved to the position at which stimulation was followed by the MEPs with the greatest amplitude and a constant shape. Trans-spinal MS was delivered using a flat round coil with an outer diameter of 114 mm. As in the case of transcranial MS, the coil was first placed at the level of L5–S1 segments of the lumbar spine, and then its position was adjusted to achieve the largest MEPs (Figure 3B). The stimulation area was picked in such a way that MEP amplitudes were generally stable, which means MEPs had a constant shape and their amplitudes were similar. Motor responses of soleus and gastrocnemius muscles were recorded with bipolar surface silver-chloride electrodes that were placed in the center of the muscle belly projections with a 20-mm interelectrode distance (Figure 3C). Electromyographic signals (Figure 3D) were recorded using a Viking Quest 4-channel myograph (Viasys, USA) with a 2-Hz to 10-kHz passband. The sensitivity band was 0.1 μV to 10 mV, and the input noise did not exceed 40 μV.

After obtaining proper coil positioning, we first retrieved the resting MEP thresholds. For this, we decreased or increased the stimulation magnitude in steps of 2–5% of maximal output until reaching the minimal stimulus magnitude to evoke motor responses of 20–50 μV amplitude with 50% or more probability. That magnitude was taken as a threshold (Nikitin and Kurenkov, 2003). The muscle relaxation during threshold evaluation was monitored *via* a real-time electromyogram (EMG). We then increased stimulation magnitude to 80% of maximal output during transcranial MS and to 70% during trans-spinal MS and recorded at least eight evoked responses. MEPs for transcranial and trans-spinal MS are referred to as “cortical MEPs” and “spinal MEPs,” respectively.

### 2.4. Data processing and statistical analysis

MEP data were extracted from muscle curves using the Viking Quest 11.1 software, and the muscle response's raw latency and amplitude values for each stimulus were obtained. For each subject, we evaluated MEP thresholds, mean peak-to-peak amplitudes at chosen stimulation magnitudes, and mean latencies (the time interval between the MS artifact and the first deflection of the muscular response from the EMG baseline) at four registration points, namely, baseline (average of four baseline points; outliers were excluded), R+0, R+3, and R+6. We also evaluated CMCT, which was calculated with the following formula: CMCT = cortical MEPs latency—spinal MEPs latency.

For demonstration purposes, threshold, amplitude, and latency values were presented as mean ± SEM of percent changes from baseline, which was taken as zero. Statistical analysis was performed with the GraphPad Prism 8 software. Data normality was assessed using the Kolmogorov-Smirnov test. For normally distributed datasets, mean values were compared using a repeated measures one-way ANOVA with a *post-hoc* Tukey's multiple comparisons test. In cases where data was not normally distributed, we used the Friedman test with a *post-hoc* Dunn's multiple comparisons test. The data were assumed to be statistically significant at p < 0.05.

## 3. Results

### 3.1. Goldoferrin C vs. control

The group of participants who got the food supplement “Goldoferrin C” during the experiment did not statistically differ from the control group in terms of MEP thresholds, amplitudes, and latencies. Thus, two groups were combined. The following are the overall results for 16 volunteers.

### 3.2. MEP thresholds

**Figure 4:**
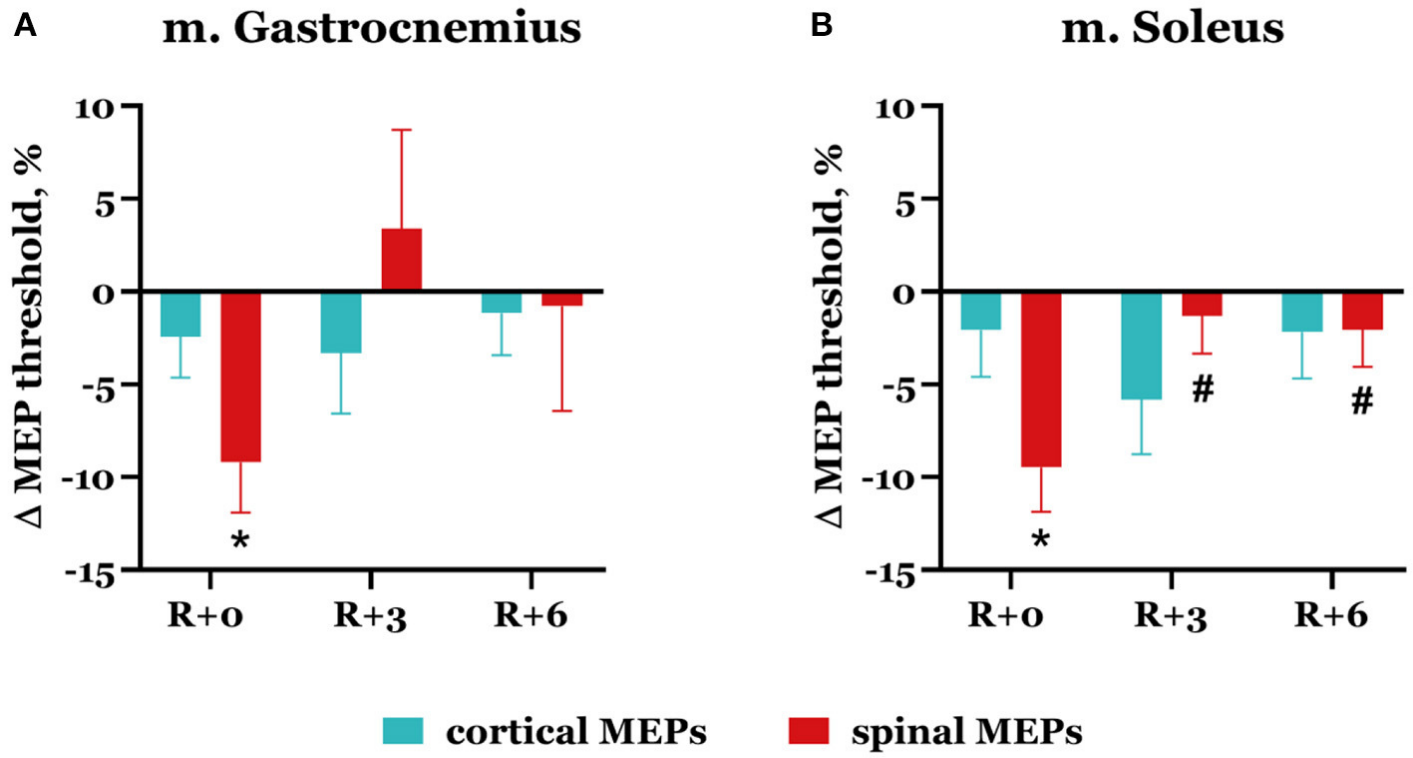
Percent changes from baseline of cortical (blue bars) and spinal (red bars) MEP thresholds in gastrocnemius **(A)** and soleus **(B)** muscles. Data are presented as mean ± SEM; baseline values are taken as zero. R+0—the day of DI completion; R+3, R+6−3rd and 6th days after DI. *p < 0.05 vs. baseline; ^#^*p* < 0.05 vs. R+0.

On the day of DI completion, spinal MEP thresholds decreased compared with baseline in both studied muscles (Figure 4): by 9.2 ± 2.7% in m. gastrocnemius (*p* = 0.042) and by 9.5 ± 2.4% in m. soleus (*p* = 0.017). During the recovery period, threshold values returned to baseline. Cortical MEP thresholds slightly decreased after DI completion: by 2.4 ± 2.2% in m. gastrocnemius and by 2.1 ± 2.5% in m. soleus. Interestingly, the biggest decrease was observed in the soleus muscle on the 3rd day after DI completion and reached 5.8 ± 2.9%.

### 3.3. MEP amplitudes

**Figure 5:**
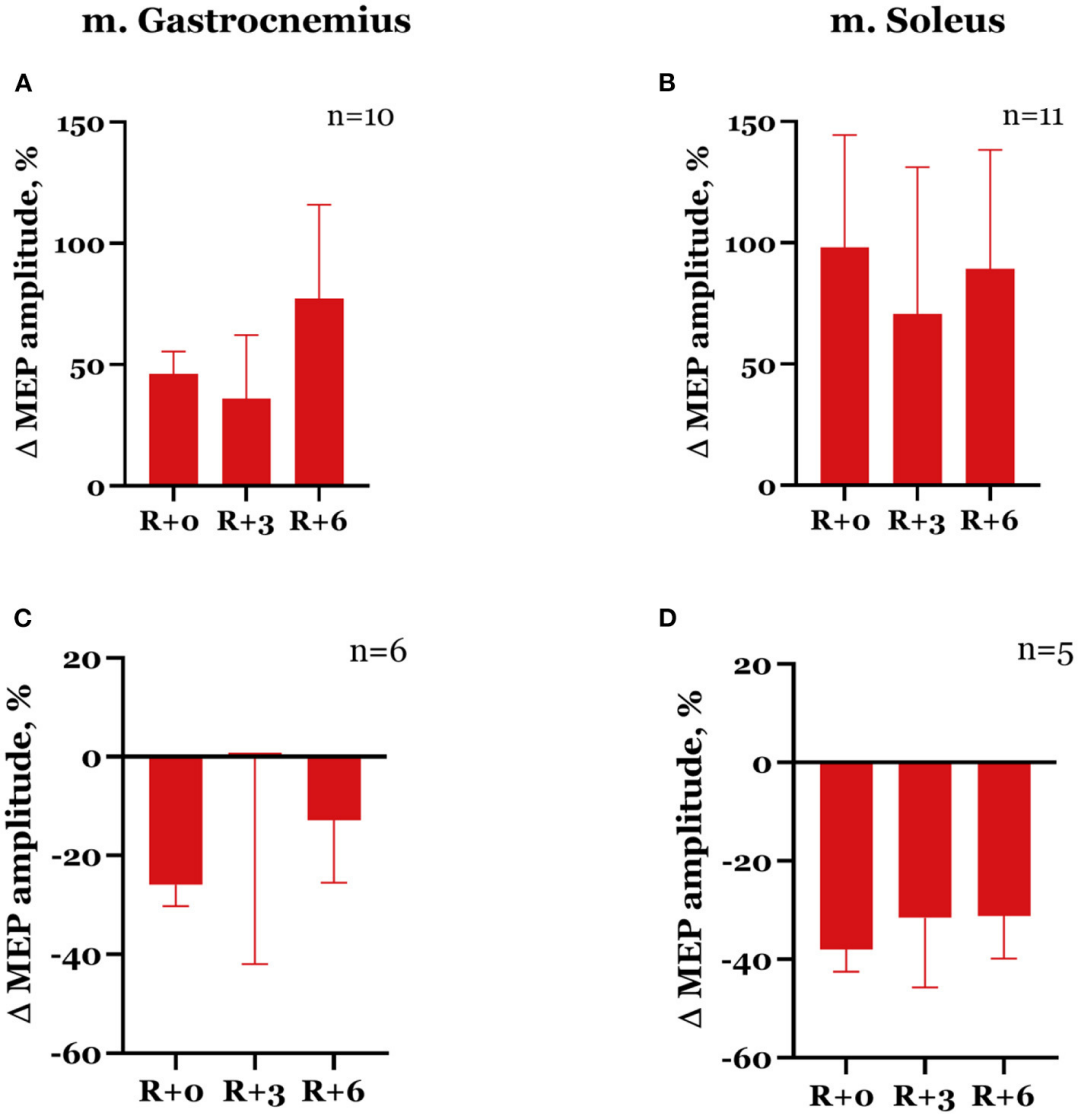
Percent changes from baseline of spinal MEP amplitudes in gastrocnemius **(A, C)** and soleus **(B, D)** muscles. Data are presented as mean ± SEM; baseline values are taken as zero. R+0—the day of DI completion; R+3, R+6−3rd and 6th days after DI.

The dynamics of spinal MEP amplitude changes during the experiment differed between the subjects. More specifically, seven subjects showed an increase in spinal MEP amplitudes after DI completion in both studied muscles. The other seven subjects had an amplitude increase in one muscle but a decrease in another one. Two subjects showed a decrease in amplitudes in both muscles. After DI, spinal MEP amplitudes increased in the gastrocnemius muscle in 10 subjects overall by 46.1 ± 9.3% (Figure 5A) and the soleus muscle in 11 subjects by 98.0 ± 46.5% (Figure 5B). For six participants, amplitudes decreased by 25.9 ± 4.3% in the gastrocnemius muscle (Figure 5C), and for five participants, by 37.8 ± 4.5% in the soleus muscle (Figure 5D). These effects were more prominent in the soleus muscle. Generally, a degree of increase in amplitude was greater than a decrease. The described changes persisted in the recovery period, although by the 3rd day after DI, amplitude values had partially returned to baseline (Table 1). None of these changes were statistically significant.

**Table 1:** MEP amplitudes mean ± SEM percent changes from baseline.

	**Gastrocnemius muscle**		**Soleus muscle**	
	**Spinal MEPs**	**Cortical MEPs**	**Spinal MEPs**	**Cortical MEPs**
R+0	46.1 ± 9.3 (*n* = 10)	81.5 ± 24.7 (*n* = 8)	98.0 ± 46.5 (*n* = 11)	31.7 ± 10.3 (*n* = 7)
	−25.9 ± 4.3 (*n* = 6)	−19.8 ± 5.3 (*n* = 8)	−37.9 ± 4.5 (*n* = 5)	−39.9 ± 7.7 (*n* = 9)
R+3	36.0 ± 26.2 (*n* = 10)	38.9 ± 14.8 (*n* = 8)	70.7 ± 60.5 (*n* = 11)	44.2 ± 18.8 (*n* = 7)
	−0.4 ± 41.6 (*n* = 6)	−11.5 ± 22.6 (*n* = 8)	−31.6 ± 14.1 (*n* = 5)	−10.5 ± 24.6 (*n* = 7)
R+6	77.2 ± 38.7 (*n* = 10)	51.9 ± 13.4 (*n* = 8)	89.2 ± 49.1 (*n* = 11)	30.5 ± 14.6 (*n* = 7)
	−12.8 ± 12.6 (*n* = 6)	−19.3 ± 5.5 (*n* = 8)	−31.2 ± 8.7 (*n* = 5)	−16.5 ± 14.5 (*n* = 7)

In the group of nine subjects that showed a decrease in spinal MEP amplitudes in one or both muscles, only one participant had an increase in spinal MEP threshold after DI completion, and only in the soleus muscle. It is worth noting that the spinal MEP amplitudes of this participant varied greatly even in the baseline studies. Such variability was also observed in another participant, but only about spinal MEP amplitudes in the soleus muscle. For the rest of this group (seven subjects), spinal MEP amplitudes in baseline sessions were mainly stable, and a common decrease in spinal MEP thresholds was observed (Figure 4).

**Figure 6:**
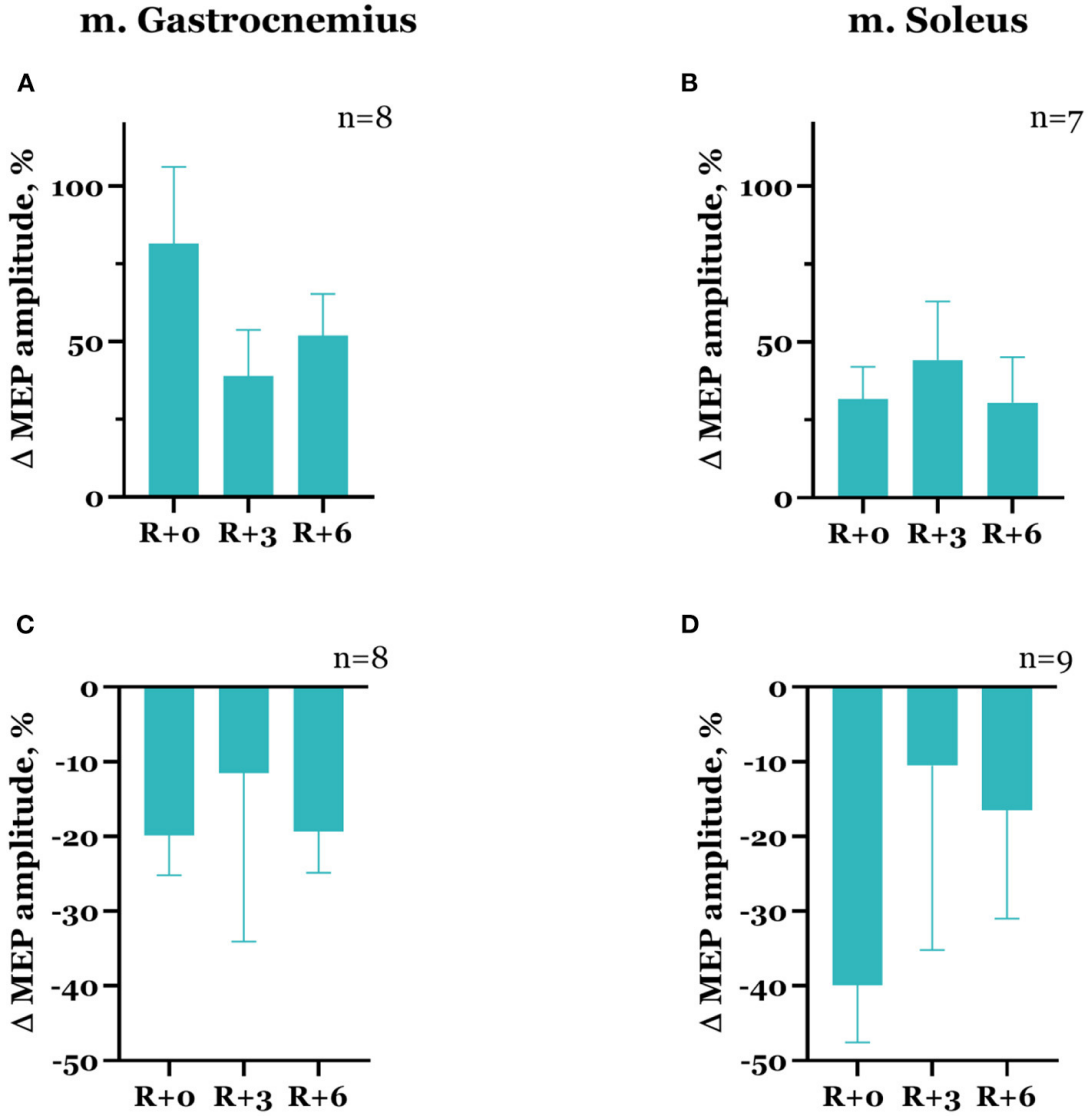
Percent changes from baseline of cortical MEP amplitudes in gastrocnemius **(A, C)** and soleus **(B, D)** muscles. Data are presented as mean ± SEM; baseline values are taken as zero. R+0—the day of DI completion; R+3, R+6−3rd and 6th days after DI.

In the majority of cases, the decrease in spinal MEP amplitude was accompanied by a decline in cortical MEP amplitude after DI completion. Although there were exceptions, specifically, one of the two participants that showed a decrease in spinal MEP amplitudes in both muscles had an increase in cortical MEP amplitudes in both muscles compared with baseline. Moreover, for some of the subjects, an increase in spinal MEP amplitude was coupled with a decrease in cortical MEP amplitude. In total, cortical MEP amplitudes increase after DI was observed in 10 subjects, of whom 6 exhibited a decrease in spinal MEP amplitudes. In the gastrocnemius muscle, cortical MEP amplitudes increased by 81.5 ± 24.7% in 8 participants (Figure 6A), and in the soleus muscle, by 31.7 ± 10.3% in 7 participants (Figure 6B). The decrease in cortical MEP amplitudes in gastrocnemius muscle was observed in 8 participants and reached 19.8 ± 5.3% (Figure 6C); in the soleus muscle, it was observed in 9 participants, reaching 39.9 ± 7.7% (Figure 6D). Cortical MEP amplitudes decreased in both muscles in 7 subjects. Interestingly, as in the case of spinal MEPs, the 6th day of the recovery period was marked by a repeated increase or decrease of cortical MEP amplitudes, even if on the 3rd day after DI amplitudes partially returned to baseline (Figures 6A, C, D, Table 1). All the described changes were not statistically significant.

It should be noted that cortical MEP characteristics varied more in baseline studies compared with spinal MEP characteristics. For instance, instability of the cortical MEP threshold or amplitude values was observed in 10 subjects. The cases of cortical MEP amplitudes increasing on the day of DI completion compared with baseline were more often accompanied by a decrease in cortical MEP thresholds, whereas when amplitudes decreased, thresholds predominantly did not differ from baseline.

### 3.4. MEP latencies and CMCT

**Figure 7:**
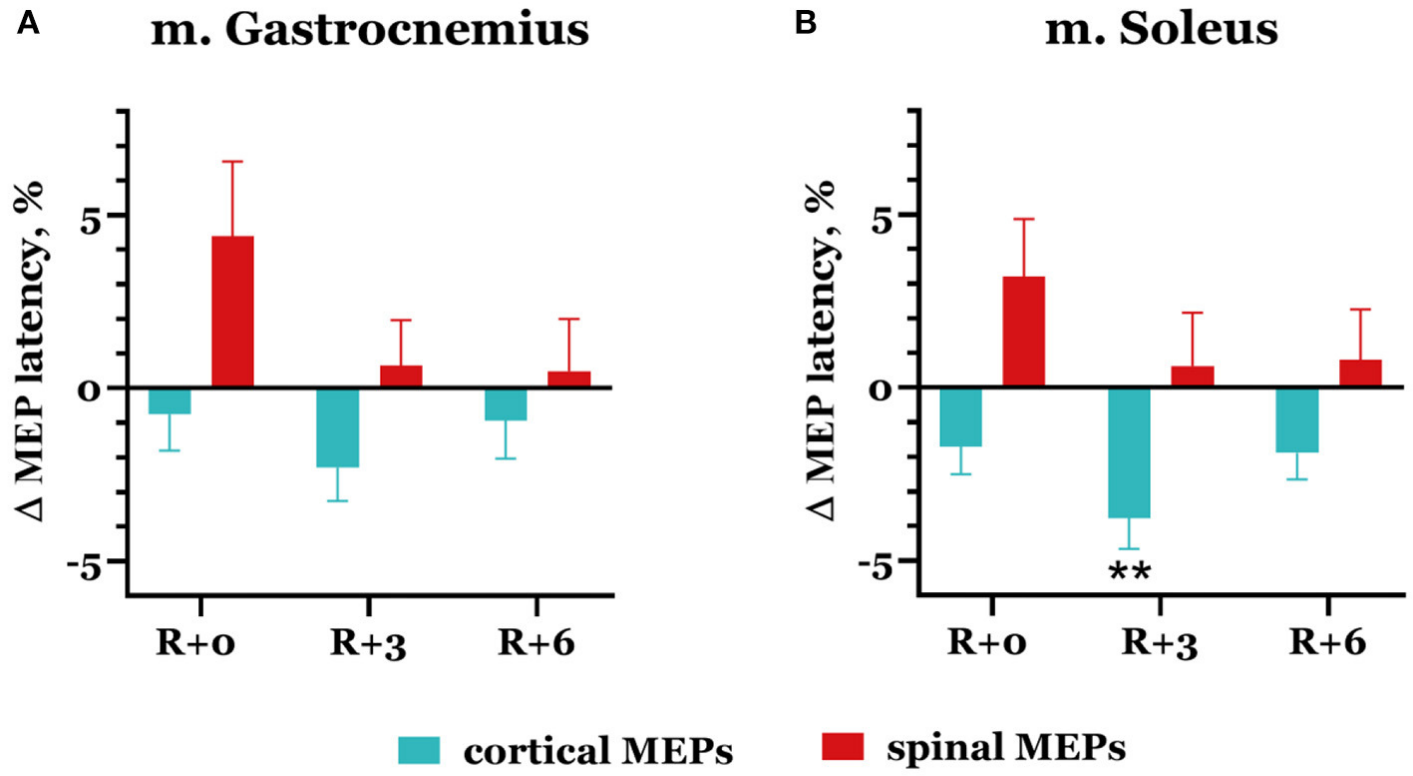
Percent changes from baseline of cortical (blue bars) and spinal (red bars) MEP latencies in gastrocnemius **(A)** and soleus **(B)** muscles. Data are presented as mean ± SEM; baseline values are taken as zero. R+0—the day of DI completion; R+3, R+6−3rd and 6th days after DI. **p < 0.01 vs. baseline.

Spinal MEP latencies increased after DI in both studied muscles: by 4.39 ± 2.2% in m. gastrocnemius and by 3.22 ± 1.65% in m. soleus (Figure 7), but these changes were not significant. Cortical MEP latencies, on the contrary, slightly decreased in the same experimental session, reaching 0.75 ± 1.06% and 1.72 ± 0.79% in the gastrocnemius and soleus muscles, respectively. By the third day of the recovery period, spinal MEP latencies returned to baseline; they averaged out at 0.66 ± 1.31% in the gastrocnemius muscle and at 0.62 ± 1.55% in the soleus muscle. At the same time, cortical MEP latencies continued to decrease: their mean values were −2.29 ± 0.97% and −3.78 ± 0.88% lower than baseline (*p* = 0.0044) in the gastrocnemius and soleus muscles, respectively.

**Figure 8:**
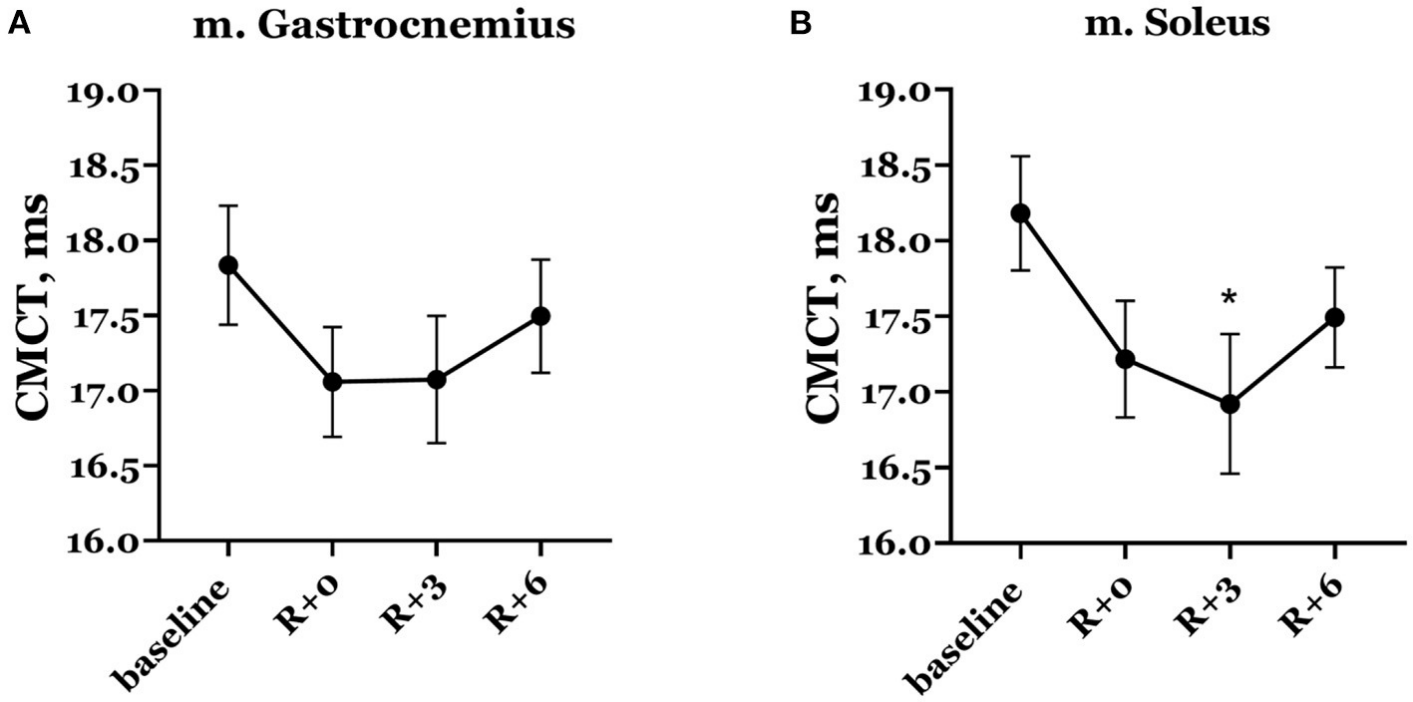
Central motor conduction time in gastrocnemius **(A)** and soleus **(B)** muscles. Data are presented as mean ± SEM. R+0—the day of DI completion; R+3, R+6−3rd and 6th days after DI. *p < 0.05 vs. baseline.

Naturally, mean CMCT decreased on the day of DI completion compared with baseline (Figure 8). In baseline studies, the mean CMCT in the group was 17.83 ± 0.40 ms in the gastrocnemius muscle and 18.18 ± 0.38 ms in the soleus muscle. On the day of DI completion, CMCT was 17.06 ± 0.37 ms and 17.22 ± 0.38 ms in the gastrocnemius and soleus muscles, respectively, although this decrease was not statistically significant. A further decrease observed in the soleus muscle on the 3rd day of the recovery period showed statistical significance compared with baseline: CMCT was 16.92 ± 0.46 ms (*p* = 0.019).

## 4. Discussion

### 4.1. General effects

After 5-day DI, we observed a decrease in MEP thresholds in gastrocnemius and soleus muscles (Figure 4), which was significant for trans-spinal MS, and a decrease in CMCT (Figure 8) in women. However, the changes in spinal and cortical MEP amplitudes in both studied muscles were ambiguous after DI completion (Figures 5, 6).

The decrease in MEP thresholds for both trans-spinal and transcranial MS and the increase in spinal MEP amplitudes in the majority of the group reflect the phenomenon of hypogravitational hyperreflexia (Kozlovskaya et al., 1988), which develops in the microgravity environment. Previously, hypogravitational hyperreflexia development on the spinal level was shown in similar immersion experiments: 5-day DI with the participation of male volunteers (Nosikova et al., 2021b) and 3-day DI with the participation of female volunteers (Nosikova et al., 2021a). There is also evidence of an increase in spinal excitability in the model of unilateral lower limb suspension (Clark et al., 2006, 2007). The majority of researchers who studied spinal reflexes in humans and animals (rats) under the conditions of SF or model experiments noted a decrease in reflex thresholds and an increase in their amplitudes (Egawa et al., 2000; Kornilova and Kozlovskaya, 2003; Zakirova et al., 2015; Tomilovskaya et al., 2019). However, the observed tendency toward a decrease in cortical MEP thresholds may suggest that hyperreflexia develops not only on the spinal level but also on the cortical level. The decrease in CMCT while spinal MEP latencies increased (Figure 7) suggests that corticospinal conductibility also increases after DI.

It is worth noting that in TMS studies conducted in other ground-based models of microgravity, there are inconsistencies in MEP characteristics' dynamics. For instance, in the parabolic flight model, an increase in cortical MEP amplitudes (Davey et al., 2004) and a decrease in thresholds (Badran et al., 2020) were revealed, which the authors, among other things, associated with an increase in corticospinal excitability. An increase in excitability was also observed after 10 days of lower limb immobilization (Roberts et al., 2007), although there were no significant changes in the evoked response thresholds. In contrast, the study in the 90-day bedrest model showed a decrease in corticospinal excitability immediately after the bedrest period ended (Roberts et al., 2010). Less research in this field and predominantly small sample sizes do not allow us to draw a firm conclusion about what may cause the described differences.

The high variability of cortical MEP characteristics in our study is possibly caused by the functional state of the subjects during experimental sessions. It is known that during the transition from wakefulness to deep sleep, corticospinal excitability decreases, which manifests in a decrease in MEP amplitudes and an increase in MEP thresholds and latencies (Grosse et al., 2002; Avesani et al., 2008). When experiencing the procedure for the first time, many participants were alert and even excited, which often resulted in higher MEP amplitudes and lower thresholds in the first session compared to subsequent sessions. On the next visits, the subjects already familiar with the procedure were calmer and more relaxed; moreover, in the sessions conducted after DI completion, several participants reported being sleepier than before DI, which could affect the obtained results.

### 4.2. Comparison of male and female groups

Previously, we conducted a similar experiment with female volunteers participating in 3-day DI (Nosikova et al., 2021a). In that work, a significant decrease in spinal MEP thresholds and a small decrease in CMCT were also observed after DI. Thresholds' decrease in the gastrocnemius muscle was twice as large as in this study; in the soleus muscle, the decrease was slightly less. It is also important to note that threshold values' variability (SEM) was substantially larger in the 3-day DI study (almost four times as large), although it was probably caused by the small sample size: only six female volunteers participated in the 2021 study. The decrease in CMCT was similar in both 3-day and 5-day DI studies, but during the recovery period after 3-day DI, CMCT increased greatly as opposed to the current results. This may suggest that longer DI exposure leaves a more persistent effect on corticospinal conductibility. After 3-day DI, mean spinal MEP amplitudes increased, though the increase was not statistically significant; moreover, the amplitudes were marked by high variability. In this study, there was an increase in spinal MEP amplitudes in the majority of the subjects; besides, the increase in amplitudes was more pronounced in the group than the decrease. As in the case of spinal MEP thresholds, spinal MEP amplitudes' increase in the gastrocnemius muscle was more than twice as large after 3-day DI compared to 5-day DI. In the soleus muscle, on the contrary, the increase in amplitudes was ~20% less in the previous study. Cortical MEP characteristics in the present work and the 2021 study were different. After 3-day DI, cortical MEP amplitudes decreased while thresholds increased. In the present study, the changes in cortical MEP amplitudes after DI completion were ambiguous, and the thresholds tended to decrease. These differences may derive from both the small sample size and the different durations of DI exposure. What is more, in the previous study, we analyzed maximal MEP amplitudes, while in the present study, we analyzed amplitudes at the fixed magnetic stimuli magnitudes, which could also cause the difference in the results. The MS protocol was adapted to minimize subjects' discomfort and to increase the quality of raw data without prolonging the procedure.

The results of our study correspond with the data obtained in the 5-day DI experiment with the participation of male volunteers (Nosikova et al., 2021b). In the male group, a significant decrease in spinal MEP thresholds and a tendency to decrease in cortical MEP thresholds were also observed. In addition, maximal spinal MEP amplitudes in men were significantly increased after DI in both muscles (the increase exceeded 100%), and maximal cortical MEP amplitudes also increased, reaching the level of significance compared with baseline in the m. soleus (increased by 60%). The increase in MEP amplitudes in the female group generally was less pronounced, which may be caused not only by a different approach to amplitude analysis (we did not assess maximal amplitudes in the female group) but also by hormonal impact on the studied characteristics in women.

There are almost no TMS studies on women in ground-based conditions modeling some or other of the SF factors. The above-mentioned experiments by Roberts et al. (2007, 2010) were conducted with the participation of mixed groups, but the data of male and female volunteers were not compared. After that, the results of those experiments were conflicting. Regarding other works dedicated to studying the neuromuscular system in SF or ground-based models, in the research of the contractile properties of the leg triceps muscle before and after 120-day HDBR, a greater depth of changes in strength characteristics was shown in men than in women (Koryak, 2009). The author links it to both the changes at the muscle fiber level and the alterations in the central mechanism of voluntary movement control.

Since the women with a natural MC participated in our study, hormonal fluctuations during MC could affect the obtained results. According to the literature, evidence of the effects of sex hormones on the neuromuscular system is contradictory. Estradiol increases cortex excitability, while progesterone modulates inhibition, and the balance of these hormones affects intracortical facilitation (ICF) and inhibition (ICI): the greatest ICF is observed at the late follicular phase, when estradiol levels are high and progesterone is low, and ICI is the greatest at the luteal phase, when progesterone levels rise (Smith et al., 2002). A better performance in the manual dexterity task was shown at the mid-luteal phase of MC compared to the ovulation phase (Zoghi et al., 2015), and grip strength decreased during the early follicular phase (Weidauer et al., 2020), although the authors did not find any correlation between these measures and the changes in estradiol and progesterone levels during MC. Several other studies did not show significant differences in strength and biomechanical characteristics of leg muscles during MC (Abt et al., 2007; Ansdell et al., 2019), and there were no changes in spinal (Casey et al., 2016) or corticospinal excitability (Ansdell et al., 2019). However, there is evidence of an increase in TMS-evoked motor responses during muscle contraction on the 14th day of MC (late follicular phase) and a decrease in neuromuscular fatigability on the 21st day of MC (Ansdell et al., 2019). In our study, baseline TMS sessions were conducted at the early follicular phase; the session on the day of DI completion coincided with the ovulation phase; and the sessions in the recovery period fell within the luteal phase (Figure 2). Since the dynamics of MEP characteristics in our study largely correlated with the data obtained in the experiment with the participation of men (Nosikova et al., 2021b), hormonal fluctuations during MC probably did not significantly affect the recorded values compared with the effects of immersion. Nevertheless, high MEP amplitudes' variability and a tendency of amplitude values on the 6th day of the recovery period to return to the state of the day of DI completion (Figures 5, 6) in women may be associated with both the hormonal level fluctuations and individual aspects of the nervous system's adaptation to the experimental conditions.

### 4.3. Effects of exposure duration

The described differences in the results of two immersion experiments with the participation of women may derive from DI duration. It is possible that after a longer exposure to support withdrawal, CNS reactions and corticospinal excitability change, which is reflected in the characteristics of cortical MEPs: cortical MEP thresholds increased after 3-day DI and amplitudes decreased (Nosikova et al., 2021a), while in the present study, cortical MEP thresholds tended to decrease and amplitudes in roughly half the instances increased after DI completion. The duration of immersion exposure could also affect the peripheral nervous system because spinal MEP latencies slightly decreased after 3-day DI and, on the contrary, increased after 5-day DI. CMCT decreased insignificantly after 3 days of the immersion experiment, but the decrease was more prominent after 5 days of exposure to support withdrawal, and by the 6th day of the recovery period, CMCT did not completely return to baseline. Different systems of the body react to the immersion exposure at different speeds, for example, heart rate and blood pressure parameters, as well as muscle tone, change significantly in the first hours of DI, while the state of bone tissue remains stable for 7 days of DI (Navasiolava et al., 2011). It was shown that H- and M-responses characteristics differ on the 3rd and 7th day of DI; moreover, the relative amplitude of the H-response gradually increases throughout the experiment (Zakirova et al., 2015). Similarly, the size of muscle fibers gradually decreases over the course of the 7-day DI (Shenkman and Kozlovskaya, 2019). Thus, longer immersion exposure leads to more profound changes in the neuromuscular system, which may be reflected in MEP characteristics.

In particular, the increase in spinal MEP latencies after 5-day DI, compared with the slight decrease in latencies after 3-day DI, could have been affected by the changes in muscles and neuromuscular synapse activity. It is known that support afferentation withdrawal leads to fast and prominent changes in both postural and mixed postural-locomotor muscles, primarily to muscle atony and atrophy, which mostly affect slow muscle fibers. Due to the changes in the cytoskeleton, muscle fibers become less sensitive to calcium, and their contractility decreases (Shenkman and Kozlovskaya, 2019). In animal experiments, it was shown that support withdrawal results in a decrease in the postsynaptic membrane surface area covered with acetylcholine receptors, as well as a decrease in the number of receptors on the “fast” muscles' nerve endings (Tyapkina et al., 2006). Although the increase in spinal MEP latencies was not significant, and we do not know how pronounced the described molecular changes are after 5 days of DI, it is worth considering the possibility of these mechanisms affecting the evoked muscle responses. It may be noted that despite the increase in spinal MEP latencies, cortical MEP latencies and CMCT decreased after DI, which potentially indicates some compensatory and adaptive processes in the CNS. Further research is needed for a more detailed analysis of the discovered phenomena and the revealing of the mechanisms behind them.

## 5. Conclusion

The results of our study show that exposure to 5-day DI in women led to the development of hyperreflexia. This phenomenon was manifested in the decrease in MEP thresholds, the predominant increase in spinal MEP amplitudes, and the decrease in CMCT. These effects correspond with the results obtained in similar experiments with the participation of men. The decrease in MEP thresholds and the increase in MEP amplitudes indicate an increase in both spinal and corticospinal excitability due to the DI exposure, while the decrease in CMCT may suggest an increase in corticospinal conductibility. The observed variability of MEP amplitudes could be caused by several factors, and more research is needed to identify its origin. The described changes in MEP characteristics during the experiment are within the limits of the physiological norm, though they still might affect astronauts' performance and should be considered for further studies.

## Data availability statement

The raw data supporting the conclusions of this article will be made available by the authors, without undue reservation.

## Ethics statement

The studies involving humans were approved by the Bioethical Commission of the Institute of Biomedical Problems of the Russian Academy of Sciences. The studies were conducted in accordance with the local legislation and institutional requirements. The participants provided their written informed consent to participate in this study.

## Author contributions

IN and AR collected data, performed data analysis, and wrote the draft of the manuscript. VK contributed to the technical support. ET revised the manuscript and was a supervisor of the study.
